# Recognizable neonatal clinical features of aplasia cutis congenita

**DOI:** 10.1186/s13052-020-0789-5

**Published:** 2020-02-18

**Authors:** Ingrid Anne Mandy Schierz, Mario Giuffrè, Antonello Del Vecchio, Vincenzo Antona, Giovanni Corsello, Ettore Piro

**Affiliations:** 1 Neonatal Intensive Care Unit, AOUP “P. Giaccone” Department of Health Promotion, Mother and Child Care, Internal Medicine and Medical Specialties “G. D’Alessandro”, University of Palermo, Via Alfonso Giordano n. 3, 90127 Palermo, Italy; 2 Neonatal Intensive Care Unit, “Di Venere” Hospital Department of Women’s and Children’s Health, University of Bari, Bari, Italy

**Keywords:** Retrospective study, Scalp defect, Meningomyelocele, Abdominal wall defect, Junctional epidermolysis bullosa

## Abstract

**Background:**

Aplasia cutis congenita (ACC), classified in nine groups, is likely to be underreported, since milder isolated lesions in wellbeing newborns could often be undetected, and solitary lesions in the context of polymalformative syndromes could not always be reported. Regardless of form and cause, therapeutic options have in common the aim to restore the deficient mechanical and immunological cutaneous protection and to limit the risk of fluid leakage or rupture of the exposed organs. We aimed to review our institutional prevalence, comorbidities, treatment and outcome of newborns with ACC.

**Methods:**

We conducted a retrospective study including all newborns affected by ACC and admitted at the University Mother-Child Department from October 2010 to October 2019. Anthropometric and clinical characteristics of ACC1 versus a non-isolated ACC group were analyzed.

**Results:**

We encountered 37 newborns, 16 with ACC1 versus 21 with non-isolated ACC. The incidence rate of 0.1% in ACC1 was higher than expected, while 19% of cases showed intrafamilial autosomal dominant transmission. Higher birth weight centile, though lower than reference population, being adequate for gestational age, normal Apgar score and euglycemia characterizing ACC1 resulted associated to a rapid tissue regeneration. Non-isolated ACC, in relation to concomitant congenital anomalies and higher prematurity rate, showed more surgical and medical complications along with the risk of neonatal death. Specifically, newborns with ACC4 were characterized by the frequent necessity of abdominal wall defect repair, responsible for the occurrence of an abdominal compartment syndrome.

**Conclusion:**

Prompt carefully assessment of the newborn with ACC in order to exclude concomitant other congenital malformations, provides clues to the underlying pathophysiology, and to the short-term prognosis. Family should be oriented toward identification of other family members affected by similar pathology, while obstetric history should exclude initial multiple pregnancy with death of a co-twin, placental anomalies and drug assumption. Molecular-genetic diagnosis and genetic counseling are integrative in individualized disease approach.

## Background

Aplasia cutis congenita (ACC) is a clinical neonatal finding of absence or defects involving the skin and encompassing heterogeneous disorders of various etiologies and severity. It can occur anywhere on the body, but most commonly presents as a small erythematous-ulcerated or scar-like alopecic ectodermal lesion on the scalp vertex.

The incidence of ACC is about 0.3‰ of live births [1]. Thus, ACC is likely to be underreported, since milder isolated lesions in wellbeing newborns could often be undetected. Moreover, solitary lesions in the context of polymalformative syndromes could not always be reported.

ACC may occur as an isolated cutaneous malformation (solitary or multiple defects, involving one or more skin districts) or in association with multiple malformations of others organ systems. Therefore, ACC may represent an index of suspicion to rule out developmental anomalies or complex syndromes, inherited skin fragility or destructive consequences in otherwise normally developed skin [1, 2].

The classification of ACC into nine groups on the basis of location and pattern of the skin defect, presence of associated abnormalities, and mode of inheritance, has been recently updated by molecular-genetic diagnoses [2, 3] (Table 1). Even the non-syndromic form of ACC1 (MIM #107600) has been found to be associated to a gene defect (*BMS1* gene) [5], resembling a ribosomopathy slowing skin morphogenesis by p21-mediated cell cycle arrest. Alterations in other regulator proteins resulting in enhanced cell migration rate and reduced cell proliferation in ACC are described, p.e. for Adams-Oliver syndrome [6].

**Table 1:** Classification for Aplasia cutis congenita (ACC), modified [2–4]

	localization	Associated malformations	Mode of inheritance
1-nonsyndromicACC	Scalp, usually paramedian vertex	Occasionally other ectodermal anomalies inclusive supernumerary nipples	AD (*BMS1*)/sporadic
2-ACC with limb abnormalities	Midline scalp	• Transverse limb defects (Adams-Oliver syndrome) • Cardiovascular anomalies: congenital heart defects (e.g. Fallot), Cutis marmorata teleangectasica congenita (19%), incomplete retinal vascularization, hemangioma, hepatoportal sclerosis, stenoses of pulmonary and intestinal veins, placental vascular abnormalities, woolly hair • ocular and CNS malformations	AD *(gens ARHGAP31, DLL4, NOTCH1, RBPJ)/*AR *(gens DOCK6, EOGT)*
3-ACC with epidermal nevi	Scalp, symmetrical distribution	• conjuntival and corneal limbal dermoids (oculo-ectodermal syndrome of Toriello-Lacassie-Droste) • sebaceous nevus, CNS malformations, limbal dermoids, pigmented nevus (SCALP syndrome) • didymosis aplasticosebacea	sporadic
4-ACC overlying embryologic malformation	Scalp, trunk	meningomyelocele, porencephaly, leptomeningeal angiomatosis, cranial stenosis, spinal dysraphism, gastroschisis, omphalocele, bladder exstrophy	Depending on underlying causes
5-ACC with *fetus papyraceus* or placental infarcts	Scalp, trunk, extremities (multiple, symmetric areas, often stellate)	Single umbilical artery, fetal growth restriction, limb anomalies, amniotic bands	Sporadic
6-ACC of extremities associated to epidermolysis bullosa	Predominantly extremities	Blistering of skin, gastrointestinal atresies, anomalies of ears, kidneys and skeleton, artrogryposis, nail deformities (Bart syndrome and other dystrophic forms of epidermolysis bullosa)	AD/AR/sporadic
7- ACC of extremities not associated to epidermolysis bullosa	Extremities (extensor areas)	–	AD/AR
8-ACC caused by teratogens	Scalp (methimazole), diffuse (varicella, herpes simplex)	Anorectal malformation (methimazole), hepatosplenomegaly and other signs of intrauterine infections (varicella, herpes simplex), hypertrophic cardiomyopathy in children of diabetic mother	–
9-ACC associated with malformation syndromes	Craniofacial, trunk	• trisomy 13 • 4p deletion syndrome • Xp22.2 deletion syndrome • Xp22.31 ectodermal dysplasia • TP63 associated syndromes (Ectrodactyly, ectodermal dysplasia, and cleft lip/palate syndrome 3; Hay-Wells syndrome; Acro-dermato-ungual-lacrimal-tooth syndrome) • bitemporal ACC (Brauer, Brauer-Setleis and Setleis syndromes) • Kabuki syndrome • intestinal malabsorption (Johanson-Blizzard syndrome) • intestinal lymphangiectasia (Bronspiegel-Zelnick syndrome) • oculocerebrocutaneous syndrome with cerebellar anomalies (Delleman syndrome) • scalp-ear-nipple syndrome (Finlay-Marks syndrome) • Knobloch syndrome (retinal detachement, occipital primary encephalocele) • amniotic band disruption complex	Depending on specific syndrome: AD/AR/X-linked/sporadic

Life-threatening complications must be prevented with timely multidisciplinary support not only in case of large lesions associated with defects in the underlying bone and dura mater or associated with embryologic malformations (meningomyelocele, porencephaly, leptomeningeal angiomatosis, cranial stenosis, spinal dysraphism, gastroschisis, omphalocele or bladder exstrophy) [2, 7].

Regardless of form and cause, therapeutic options have in common the aim to restore the deficient mechanical and immunological cutaneous protection and to limit the risk of fluid leakage or rupture of the exposed organs [7, 8]. Small lesions without exposed organs usually heal spontaneously over a few weeks, even when the bone is involved. Conservative treatment of larger scalp defects includes the use of adhering or non-adhering dressings containing hydrogel, emollients, silver or topical agents. Newly proposed treatments as topical ozone therapy may promote also the osteogenic potential of the bone surrounding the defect [7]. Emergency surgical treatment options in case of defect sizes > 15 cm^2^ include coverage with allogenic dermis, full-thickness or split-thickness skin grafts, cultured keratinocyte autografts, free or local flaps, and early reconstructive cranioplasty. Residual scars, alopecies, or contractures take advantage by a second-surgery revision.

The prognosis for small self-healing scalp lesions is excellent. The estimated mortality rate among patients with larger scalp ACC type 1 and 2 is high as 14% [7]. Because there are only individual reports for the other forms, it is difficult to calculate a mortality rate or to give an individualized prognosis. Therefore, we aimed to review our institutional prevalence, comorbidities, treatment and outcome of newborns with ACC.

## Methods

After approval from our Hospital Ethic Committee, we performed a retrospective study including all newborns affected by ACC and admitted at the Mother-Child Department of University Hospital of Palermo from October 2010 to October 2019. Neonatal anthropometric data, perinatal clinical characteristics, comorbidities, healing outcome and survival rate were collected from charts and analyzed. Written informed parental consent was obtained at admission for use of patient’s data.

Statistical analyses were performed by the open source statistical R 3.04.0 software (R Development Core Team, Vienna, Austria), and the significance was defined as *p*-value < 0.05. Anthropometric and clinical characteristics of the ACC1 versus the non-isolated ACC group were compared using Kruskal–Wallis tests for continuous variables, and Fisher’s exact tests for categorical variables.

## Results

We enrolled 37 newborns (15 Females/22 Males) and identified 16 patients (43%) with ACC1 and 21 patients with non-isolated forms (49% ACC4 and 8% ACC6). Considering only inborn patients, the incidence rate was 0.2% of all types, and 0.1% of only ACC1, while the prevalence in our neonatal care unit was 1.3 and 0.5%, respectively.

Within the ACC1 group only one patient, the only preterm < 34 weeks gestation, born from a diabetic mother, was affected by a millimetric interventricular septum defect that closed spontaneously during the hospital stay. No patient in the ACC1 group had cerebral or ocular malformations. The mean diameter of lesion was 3.1 ± 4 cm (range 0.3–10 cm). We encountered 19% of cases with intrafamilial transmission, one case through three generations, two cases through two generations. Except for the larger defect, all lesions showed a spontaneous healing. The larger defect in a male newborn, patient 1, required an emergency surgical treatment by coverage with a dermal matrix allograft, daily occlusive medications and ambulatory follow up. It healed within 2 months.

Array-comparative genomic hybridization (a-CGH) analysis was conducted in two patients with large ACC1 scalp defects. In patient 1 we identified a 19q12(30173190_30286951) duplication of 113 kb encompassing the gene *C19orf12* in the GRCh37 code, linked to neurodegeneration with brain iron accumulation 4. Since no central nervous system anomalies in proband and no neurodegenerative symptoms in relatives were present, we did not proceed with further investigations.

Patient 2 with familiar recurrence, showed multiple duplications: 10p15.1(5462228_5896539) duplication of 434 kb encompassing *NET1*, *CALML5*, *CALML3*, *ASB13*, *GDI2*, *TASOR2*; 10p14(7858350_8042996) duplication of 184 kb encompassing *TAF3*; 11q25(131110755_131257447) duplication of 146 kb encompassing *NTM*; 12q24.33(132475221_132626501) duplication of 151 kb encompassing *EP400*, *SNORA49*, *EP400NL*, *DDX51* in the GRCh37 code. Since multiple duplications involved manifold protein regulators, not described in ACC, informed parents refuted to proceed with further studies.

The most frequent pathology in the non-isolated ACC group was congenital abdominal wall defect (6 patients with gastroschisis; 8 patients with lateral, 2 with caudal, 1 with cephalic fold omphalocele). Less frequent pathologies were meningomyelocele (1 patient) and epidermolysis bullosa (1 patient with simplex type, 2 with junctional generalized severe type). All patients in the non-isolated ACC group showed a high risk of other associated congenital anomalies. Gastrointestinal malformations were the most frequent (29% congenital malformation of intestinal fixation, 19% congenital atresia and stenosis of intestine, 14% Meckel’s diverticulum), followed by 33% cardiac, 19% musculoskeletal, 19% genitourinary (epispadias/hypospadias), and 5% involving the central nervous system. Congenital inguinal hernia at admission was present in 19% newborns. In this study, no specific genetic alterations have been identified with gastroschisis, but omphalocele was associated with chromosomal anomalies in 9.5% (Trisomy 18, derivative Y chromosome) and monogenic alterations in 9.5% of cases (Noonan syndrome, Beckwith-Wiedemann syndrome). In both patients with generalized severe junctional epidermolysis bullosa (JEB-gen sev) we found homozygote known mutations in *LAMC2* gene (c.1A > G and c.667C > T, respectively).

Sixteen patients with primary hernia reduction and defect closure required assisted mechanical ventilation, nasogastric decompression and sustained intravenous fluids. Additional lateral relaxing incisions in the fascia were performed for primary closure in the case of meningomyelocele. Different operative techniques were used to stretch the abdominal wall in preparation for primary closure in abdominal wall defects. Concomitant gastrointestinal malformations were treated, in three patients by temporary enterostomy. In two patients a Silastic silo for gradual reduction of intestine was placed and subsequent defect closure by soft tissue patches (Gore-Tex) was performed. In two patients with gastroschisis, after primary closure of wall defect, an emergent decompression by silo was required owing to abdominal compartment syndrome.

Analyzing isolated and non-isolated ACC, we found similarities in maternal variables and some differences in anthropometric values, clinical features and short-term prognosis (Table 2).

**Table 2:** Anthropometric and clinical features of ACC at birth and clinical outcome at 1-month Follow-up, with *p* values

	ACC1 (*n* = 16)				Non-isolated ACC (*n* = 21)				
	Mean	SD	Median	IQR	Mean	SD	Median	IQR	*p*
Mother’s age (years)	31.0	3.7	31	5	29.9	9.5	29	8.5	*0.42610*
Gestational age (weeks)	38^+ 3^	2^+ 2^	39	2	36^+ 6^	2^+ 5^	37^+ 3^	3	*0.07217*
Parity	1.5	0.5	1.5	1	1.6	0.6	1.5	1	*0.88460*
Gender (F/M)			1				0.62		*0.51950*
Cesarean section (%)			69				67		*0.74750*
Prematurity (%)			18.8				47.6		*0.09118*
Prematurity < 34 weeks (%)			6.3				19.0		*0.36410*
Weight (g)	3023	403	3015	522.5	2442	697	2645	1098	***0.00903***
Weight centile	42.3	24.9	37	37	24.3	24.0	13	23.5	***0.01761***
SGA (< 10° centile, %)			6.3				33.3		***0.03923***
Length centile	32.8	25.6	36	40	21.1	22.9	14.5	28.3	*0.23630*
Head circumference centile	42.3	26.4	37	28.5	42.7	35.3	46	48	*0.89670*
Apgar 1’	8.9	1.0	9	0.5	7.3	1.6	8	1.5	***0.00057***
Apgar 5’	9.5	0.8	10	1.0	9.0	1.1	9	2	*0.10580*
Glycemia at birth (mg/dl)	64.4	15.7	62	27	96	17	101	16.5	*0.05271*
Calcemia (mg/dl)	9.4	1.1	9.6	1.3	8.7	1.2	8.7	2.3	*0.16530*
Parenteral nutrition (days)	0.5	1.9	0	0	28.9	41.2	17	24	***0.00004***
Hospital stay (days)	8.8	6.5	7.5	8.8	41.3	37.5	38	38	***0.00490***
Good outcome (%)			100				67		***0.01241***

*Abbreviations*: *ACC* aplasia cutis congenita, *SGA* small for gestational age. *Significant differences are in bold*

## Discussion

In this study we evaluated prevalence, comorbidities, treatment and outcome of newborns with various forms of ACC. Prevalence of ACC1 was about 3-fold higher than previously reported [9], probably because this study was based on strict medical records review and was not limited to Hospital discharge forms, that could not include small ACC in wellbeing newborns. Unlike previous reports, we did not exclude dominant forms of isolated ACC [9]. The prognosis of self-healing scalp lesions, including the larger ones, was excellent. We do not confirm in our patients the previously high mortality rate among patients with ACC1 [7].

In our sample the predominance of non-isolated ACC, in inborn infants too, is correlated to the high level of specialization in the intensive prenatal, neonatal and surgical management of patients affected by congenital anomalies and syndromes characterizing our Department. The retrospective design allowed data collection with known outcome and prevalence estimation. Incidence and prevalence were unexpectedly high, probably because incidence data in previous studies refer to works published prior to the adoption of the latest classification which includes nine clinical variants, and not detailed since ACC has been considered as a unique clinical entity.

Analyzing isolated and non-isolated ACC, we found some similarities. Maternal variables including, age, pathological conditions and no assumption of drugs potentially affecting the fetus were similar, and no statistically significant difference between groups were found for sex, neonatal length and head circumference in relation to gestational age.

On the other hand, we found some differences in other anthropometric values and clinical features.

Higher birth weight centile, though lower than reference population, being adequate for gestational age (AGA) and normal Apgar score characterizing ACC1 resulted associated to the rapid tissue regeneration, observed in all patients including the cases with larger scalp defect. Furthermore, in this group we did not observe neonatal infections since rigorous infection-control practices, along with gentle cleansing and application of topical antibiotics, have been applied in all patients.

We registered a relative lower “care burden” measured as total parenteral nutrition, assisted mechanical ventilation, surgical intervention and length of hospital stay, as in previous report on more severe, but isolated malformation [10].

Taking into account the etiology of ACC1, since in our cases we did not identify any antiphospholipid antibody syndrome or thrombophilia during pregnancy, we could exclude a pathogenic role of vascular disruption or placental infarcts [11]. In our institution drug assumption is based on self-reporting and we do not undergo toxicological tests until the neonatal neurobehavioral examination is normal, nevertheless we cannot exclude that in some cases drug assumption have been undetected.

In case of familial recurrence of ACC1, an autosomal dominant transmission was plausible as reported in previous studies [1]. In two patients, a-CGH showed the involvement of genes that could interfere with epidermal development. The most relevant finding has been the association with *NTM* (neurotrimin) gene, recently related not only to neural cell development and survival but also to skin cell adhesion and increased risk of melanoma [12].

The normal neuro-behavioral assessment and cerebral US at discharge, reported in ACC1, have a positive prognostic value in terms of normal development.

Neonatal mortality rate has not been reported previously in non-isolated ACC group.

In non-isolated ACC we observed an increased glycemic value at birth attributable to antenatal corticosteroid treatment for the prevention of respiratory distress, owing to the relative high rate of prematurity below 34 weeks gestation.

Non-isolated ACC, in relation to concomitant congenital anomalies and higher prematurity rate, showed more surgical and medical complications along with the risk of death. Specifically, newborns with ACC4 were characterized by the frequent necessity of abdominal wall defect repair, responsible for the occurrence of an abdominal compartment syndrome [13] carrying an increased risk of multiorgan failure and neonatal death.

The two patients with ACC6 presenting with JEB-gen sev and one patient with omphalocele, despite intense treatment performed in specialized centers, died at 3 months of age, confirming the ominous prognosis reported in scientific literature [14].

Although, to our knowledge, a prolonged follow-up in ACC1 on possible skin tumorigenesis has not been performed, a prominent tumor suppression role has been demonstrated in case of increased p21 levels related to *BMS1* mutations, and responsible for prominent hypertrophic scar formation observed in ACC1 [5, 15, 16].

## Conclusions

The newborn affected by ACC should be carefully examined in order to exclude concomitant other congenital malformations affecting primarily ectoderm-derived tissues and organs. Family should be oriented toward identification of other family members affected by similar pathology, while obstetric history should exclude initial multiple pregnancy with death of a co-twin, placental anomalies and drug assumption. Since birth standard infection control measures should be applied. Individualized neonatal care and a family centered model of assistance should be developed in case of ACC concomitant to other severe malformations or JEB-gen sev in relation also to the emotional difficulties the parents must face in front of their child’s potential death. Once a final molecular-genetic diagnosis has been achieved, family should receive genetic counseling regarding the risk of recurrence.

## Data Availability

The datasets used and/or analyzed during the current study are available from the corresponding author on reasonable request.

## Abbreviations

ACC: Aplasia cutis congenita
a-CGH: Array-comparative genomic hybridization
JEB-gen sev: Generalized severe junctional epidermolysis bullosa
